# Imaging features and differentiation between benign and malignant Brenner tumors in a multi-center cohort

**DOI:** 10.3389/fonc.2026.1809247

**Published:** 2026-06-12

**Authors:** Tao Zhou, Peng Xia, Qinghai Miao, Li Li, Libin Cao, Zhongqiu Wang, Zefeng Shao, Na Duan

**Affiliations:** 1 Department of Interventional Radiology, Affiliated Hospital of Nanjing University of Chinese Medicine, Nanjing, China; 2 Department of Radiology, Affiliated Hospital of Nanjing University of Chinese Medicine, Nanjing, China; 3 Department of Radiology, Wuxi Affiliated Hospital of Nanjing University of Chinese Medicine, Wuxi, China; 4 Department of Pathology, Affiliated Hospital of Nanjing University of Chinese Medicine, Nanjing, China; 5 Department of Radiology, Obstetrics and Gynecology Hospital of Nanjing Medical University, Nanjing, China

**Keywords:** Brenner tumor, computed tomography, diagnosis, magnetic resonance imaging, ovarian neoplasms

## Abstract

**Objective:**

To explore the imaging features of histopathologically confirmed ovarian Brenner tumors (BTs) and to improve the accuracy of differential diagnosis between benign and borderline or malignant variants.

**Methods:**

A retrospective analysis was conducted on clinical and imaging data from patients with pathologically confirmed ovarian BTs, collected from five medical centers between January 2014 and December 2024. Univariate and multivariate logistic regression analyses were utilized to identify independent risk factors associated with borderline or malignant BTs.

**Results:**

Clinically, the incidence of elevated serum CA125 levels was significantly greater in the borderline/malignant group compared to the benign group (p < 0.05). Regarding imaging characteristics, variables including maximum tumor diameter, tumor composition, tumor margin, presence of papillary projections, calcification pattern, degree of enhancement, as well as CT attenuation values between the non-contrast and the arterial or venous phases, demonstrated statistically significant differences between benign and malignant tumors (p < 0.05). Tumor maximum diameter, papillary projections and venous-phase CT net enhancement were identified as independent risk factors associated with borderline/malignant BTs. The combined model showed promising discriminative ability (AUC 0.956), but this result requires extreme caution.

**Conclusion:**

In pathology-confirmed BTs, maximum tumor diameter, presence of papillary projections, and venous-phase CT net enhancement are valuable markers for differentiating benign from borderline or malignant subtypes. These features may assist in preoperative imaging assessment and risk categorization.

## Introduction

Ovarian Brenner tumors (BTs) are rare neoplasms originating from urothelial transitional cells, constituting approximately 2% to 3% of all ovarian tumors (1, 2). According to the World Health Organization classification, BTs of the female genital tract are categorized as benign, borderline, or malignant (3). The benign form is the most common, whereas borderline and malignant variants represent roughly 5% and 1% of cases, respectively (4, 5).

Benign BTs have a very favorable prognosis and do not need chemotherapy. However, because borderline and malignant forms are rare, our knowledge of their biology is limited, making it difficult to differentiate them from benign tumors before surgery. Borderline BTs carry the risk of recurrence and becoming malignant, while malignant BTs are aggressive, tend to spread, and have poor outcomes (6, 7). Since treatment plans and patient prognosis depend entirely on distinguishing these types, enhancing the accuracy of preoperative diagnosis for these uncommon tumors is crucial.

Despite these insights, the majority of existing literature on BTs is derived from case reports and studies with limited sample sizes, resulting in scarce data regarding the imaging characteristics of borderline and malignant BTs. Due to the extreme rarity of this tumor type, the present retrospective study analyzed clinical data and imaging results from computed tomography (CT) or magnetic resonance imaging (MRI) in 52 patients diagnosed with BTs. The main goal of this study is to compare the clinical and imaging characteristics of cases confirmed as benign with borderline/malignant, aiming to identify potential risk factors that could assist radiologists in suspecting borderline or malignant BTs prior to surgery, thereby improving preoperative risk assessment.

## Materials and methods

### Study participants

This study received ethical approval from the Ethics Committees of Jiangsu Provincial Hospital of Chinese Medicine and the Obstetrics and Gynecology Hospital of Nanjing Medical University, with a waiver for informed consent granted (Approval Nos. 2021NL-KS066, 2023KT-009). A retrospective collection of clinical and imaging data was conducted for 65 patients diagnosed with ovarian BTs confirmed through surgical and pathological evaluation between January 2014 and December 2024. Data were obtained from five medical institutions: Affiliated Hospital of Nanjing University of Chinese Medicine, the Obstetrics and Gynecology Hospital of Nanjing Medical University, the First Affiliated Hospital of Nanjing Medical University, the General Hospital of Tianjin Medical University, and Ruijin Hospital affiliated with Shanghai Jiao Tong University School of Medicine.

**Figure 1:**
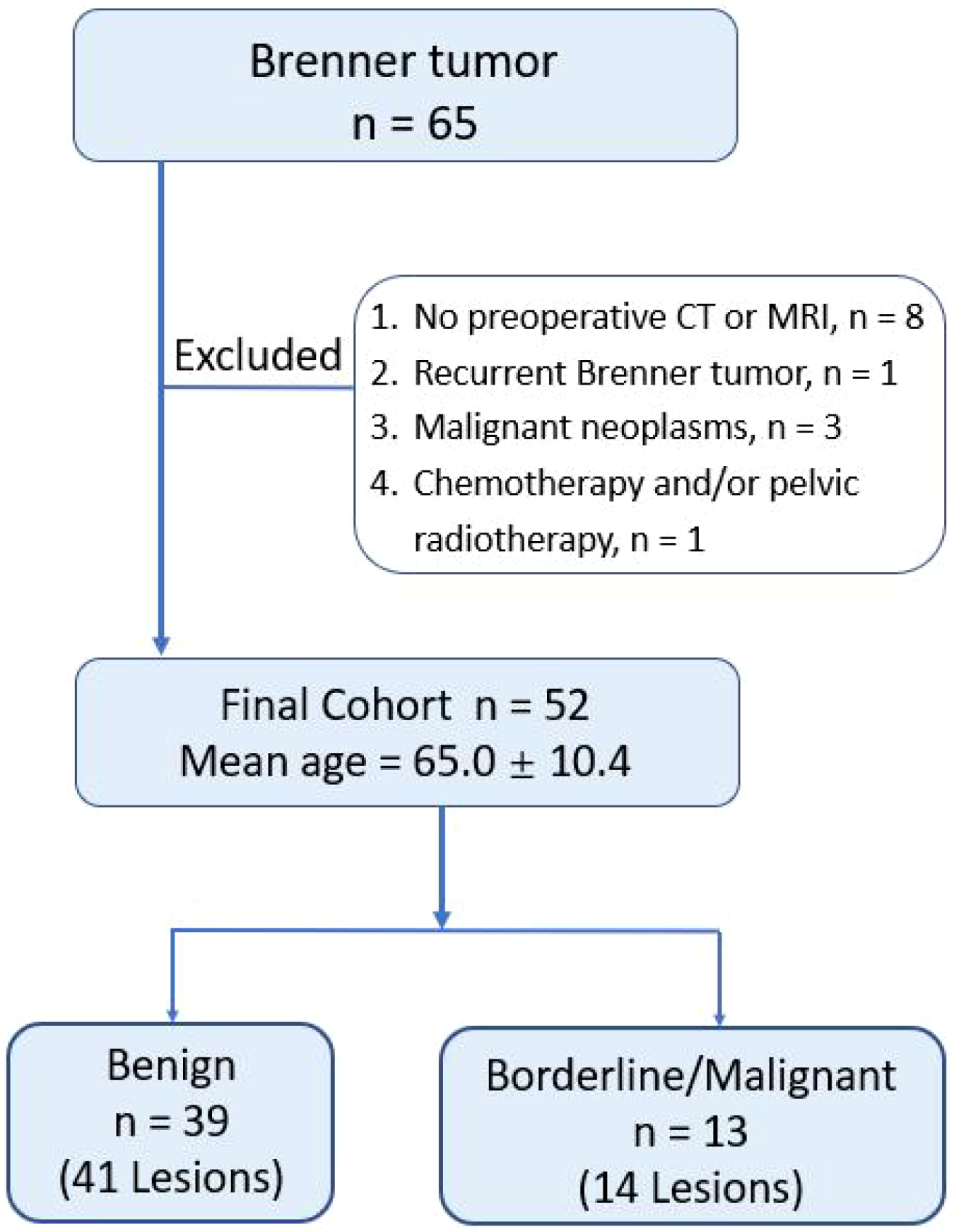
Flowchart illustrates cohort selection process.

The exclusion criteria applied were as follows: (1) absence of preoperative CT/MRI examinations; (2) presence of recurrent BT; and (3) history of any prior relevant treatment or pelvic radiotherapy. Ultimately, 55 BT lesions from 52 patients were included in the analysis, comprising 49 cases with unilateral tumors and 3 cases with bilateral tumors (**Figure 1**).

### Clinical and laboratory assessments

Clinical information was obtained, encompassing age at initial presentation, menopausal status, and reported symptoms such as abdominal pain, vaginal bleeding, and hematuria. The evaluated tumor markers comprised HE4, CA125, CA19-9, CEA, and AFP.

### CT and MRI techniques

Among the 52 patients, 36 underwent CT, 33 underwent MRI, and 17 underwent both CT and MRI prior to surgery. Of these, 29 patients received contrast-enhanced CT scans, while 20 patients underwent contrast-enhanced MRI.

CT scans were performed utilizing one of the following devices: GE BrightSpeed Elite 16-slice, GE Lightspeed Series 16-slice, GE Lightspeed VCT 64-slice (all produced by GE Healthcare), or Siemens Sensation 16-slice (Siemens Corporation). Patients were positioned supine during image acquisition. Scanning parameters included a tube voltage ranging from 100 to 120 kVp and a tube current between 200 and 400 mAs. Collimators of dimensions 0.6 × 32 mm and 1.25 × 16 mm were applied, with slice thicknesses set between 2 and 4 mm. Dynamic contrast-enhanced CT was conducted using bolus-triggered scanning techniques. An intravenous administration of approximately 80 to 100 ml of a non-ionic iodinated contrast medium—specifically Ioxaglate (320 mg/ml), Iodixanol (300 mg/ml), or Iodixanol (350 mg/ml) (supplied by GE Healthcare and General Pharmaceutical Co. Ltd)—was delivered at a rate of 1.5 to 3 ml/s. Images acquired 20 to 25 seconds post-injection corresponded to the arterial phase, whereas those obtained 60 to 70 seconds after injection represented the venous phase. Delayed phase images were captured four minutes following contrast administration. Subsequently, CT images were reconstructed with slice thicknesses ranging from 1.25 to 3 mm.

MRI examinations were conducted using scanners with magnetic field strengths of 1.5 Tesla (T) and 3.0 T, including models such as the GE Signa HDXT 1.5 T, Siemens Avanto 1.5 T, Siemens Area 1.5 T, Siemens MAGNETOM Verio 3.0 T, and Philips Ingenia 3.0 T. Each system was equipped with either torso or pelvic coils. The non-contrast pelvic MRI protocol comprised the following sequences: axial T1-weighted imaging (T1WI) with repetition time (TR) of 518–730 ms, echo time (TE) of 11–15 ms, and slice thickness of 4–5 mm; axial T1WI with fat suppression (TR 3.2–3.9 ms, TE 1.1–1.4 ms, slice thickness 2–3 mm); sagittal T2-weighted imaging (T2WI) with fat suppression (TR 4310–4500 ms, TE 90–99 ms, slice thickness 4–5 mm); axial T2WI (TR 3300–3500 ms, TE 85–95 ms, slice thickness 4–5 mm); and axial T2WI with fat suppression (TR 5100–5500 ms, TE 90–95 ms, slice thickness 4–5 mm). Diffusion-weighted imaging (DWI) was performed using b-values of 50 and 1000 s/mm² (TR/TE = 6800/80 ms; slice spacing 1 mm; slice thickness 4 mm), with apparent diffusion coefficient (ADC) maps generated automatically during post-processing. Following acquisition of unenhanced images, contrast-enhanced imaging was conducted subsequent to intravenous injection of 0.1 mmol/kg of Gadopentetate dimeglumine (BeiLu Pharmaceutical Co., Ltd.) administered at a rate of 1.5 to 2.0 ml/s. Three-phase imaging was obtained at 15 seconds, 50–60 seconds, and 3–4 minutes post-injection.

### Image analysis

This multicenter retrospective study applied a consistent standardization protocol to all imaging data before analysis to reduce variability caused by differences in equipment, scanning settings, and reconstruction techniques. The standardization process included: (1) Spatial standardization, where all images were resampled to have uniform pixel size and matrix dimensions; (2) Grayscale normalization, using Z-score transformation to standardize brightness levels, with outliers removed through percentile truncation; (3) Image quality control, where two experienced radiologists excluded images with significant artifacts or insufficient diagnostic information; and (4) Adjustment for center effects by including the study center as a covariate in the statistical analysis. These steps helped ensure the multicenter imaging data were as consistent and reliable as possible. All CT and MRI scans were archived in the Digital Imaging and Communications in Medicine (DICOM) format and subsequently imported into RadiAnt DICOM Viewer software (Version 2021.1; released June 27, 2021; available at https://www.radiantviewer.com). Two radiologists (L.C. and N.D.), with 10 and 15 years of experience in gynecological imaging diagnosis respectively, independently reviewed the images. Both reviewers were blinded to the patients’ clinical data and pathological findings. Any discrepancies between their assessments were resolved through consensus discussion.

Imaging characteristics include: tumor location, size, shape, margins (regular/irregular), unilateral/bilateral involvement, internal features (cystic if solid component <10%; solid if solid component >90%; cystic-solid if solid component between 10% and 90%), presence or absence of calcification (CT value >100 HU), and free fluid in the abdominal or pelvic cavity; For contrast-enhanced scans, the region of interest (ROI) should be selected in the area showing the most pronounced enhancement at each stage to measure CT values, avoiding areas of calcification; the average value is then calculated after measuring three different regions. When measuring ADC values, T1WI, T2WI and MRI contrast enhancement, the region of interest should be selected within the solid component of the tumor, avoiding areas of cystic necrosis. Borderline and malignant tumors were combined into a single group due to their similar clinical management and the small number of pure malignant cases, consistent with prior radiological studies on rare ovarian tumors.

### Histopathologic analysis

The gold standard for all cases was surgical pathology. To guarantee diagnostic accuracy, the following steps were taken: (a) a central review of all slides by two experienced pathologists who were unaware of the imaging results; and (b) use of the detailed 2020 World Health Organization (WHO) classification criteria for Brenner tumors, divided into these categories: Benign – well-defined clusters of transitional epithelial cells with distinctive “coffee-bean” shaped nuclei within dense fibrous tissue, showing no atypia or stromal invasion; Borderline – epithelial growth resembling low-grade papillary urothelial carcinoma with mild to moderate atypia, but without any stromal invasion; Malignant – clear malignant characteristics (high-grade atypia and increased mitotic activity) with confirmed destructive invasion of the stroma, usually developing alongside a benign or borderline component (3).

### Statistics

Statistical analysis was performed using SPSS Statistics (version 26.0; IBM Corp., Chicago, IL, USA), while GraphPad Prism (version 10.0.0 for Windows, Boston, Massachusetts USA) was used to generate graphs, presenting the differences in data between groups and the relevant analytical results. Quantitative data conforming to a normal distribution are expressed as $\bar{x}$ ± s, and comparisons between groups were performed using the two-sample t-test; quantitative data not conforming to a normal distribution are expressed as median (interquartile range), and comparisons between groups were performed using the Wilcoxon rank-sum test; qualitative data are expressed as proportions, and comparisons between groups were performed using the chi-square test. Variables with p-values less than 0.1 were included in a multivariate analysis to identify independent risk factors for borderline or malignant BTs and to differentiate these two types. The area under the curve (AUC) was calculated to assess diagnostic accuracy. The DeLong test was used to compare AUCs between models, considering p-values below 0.05 as statistically significant. To evaluate the consistency and reproducibility of imaging feature assessments, both intra- and inter-observer variability were measured. An intraclass correlation coefficient (ICC) or kappa value greater than 0.75 was interpreted as indicating excellent agreement (8).

## Results

### Clinical features

The mean age of the 39 patients diagnosed with benign BTs was 63.9 ± 11.7 years. Within this cohort, 6 patients were premenopausal, while 33 were postmenopausal. Clinical symptoms observed included vaginal bleeding in 23.1% of cases (9/39), abdominal pain in 20.5% (8/39), and hematuria in 5.1% (2/39). In contrast, the 13 patients diagnosed with borderline or malignant BT had a mean age of 70.5 ± 8.3 years, all of whom were postmenopausal. Symptomatic presentations in this group comprised vaginal bleeding in 15.4% (2/13), abdominal pain in 38.5% (5/13), and hematuria in 15.4% (2/13). Statistical analysis revealed no significant differences between the benign and borderline/malignant groups regarding age, menopausal status, or clinical symptoms. Regarding laboratory findings, elevated CA125 levels were observed in 17.9% (6/39) of patients with benign BT and in 30.8% (5/13) of those with borderline or malignant BT, with this difference reaching statistical significance. These findings are detailed in **Table 1**.

**Table 1:** Clinical features of 52 patients with benign and borderline/malignant BTs.

Clinical features	Total	Benign	Borderline/malignant	*p* value
	(n = 52)	(n = 39)	(n = 13)	
Age (year)	65.5 ± 11.2	63.9 ± 11.7	70.5 ± 8.3	0.07
Menopausal status				0.13
Premenopausal	6 (11.5)	6 (15.4)	0	
Postmenopausal	46 (88.5)	33 (84.6)	13 (100)	
Symptoms				0.63
Asymptomatic	26 (50.0)	20 (51.3)	6 (46.2)	
Bellyache	13 (25.0)	8 (20.5)	5 (38.5)	
Vaginal bleeding	11 (21.2)	9 (23.1)	2 (15.4)	
Hematuria	4 (7.7)	2 (5.1)	2 (15.4)	
Tumor markers				
CA199 (+)	15 (28.8)	10 (25.6)	5 (38.5)	0.07
CA125 (+)	11 (21.2)	6 (17.9)	5 (30.8)	**0.03**
AFP (+)	5 (9.6)	4 (10.3)	1 (7.7)	0.47
CEA (+)	4 (7.7)	2 (5.1)	2 (15.4)	0.13

Bold values indicate statistical significance (p < 0.05).

### Imaging findings

**Figure 2:**
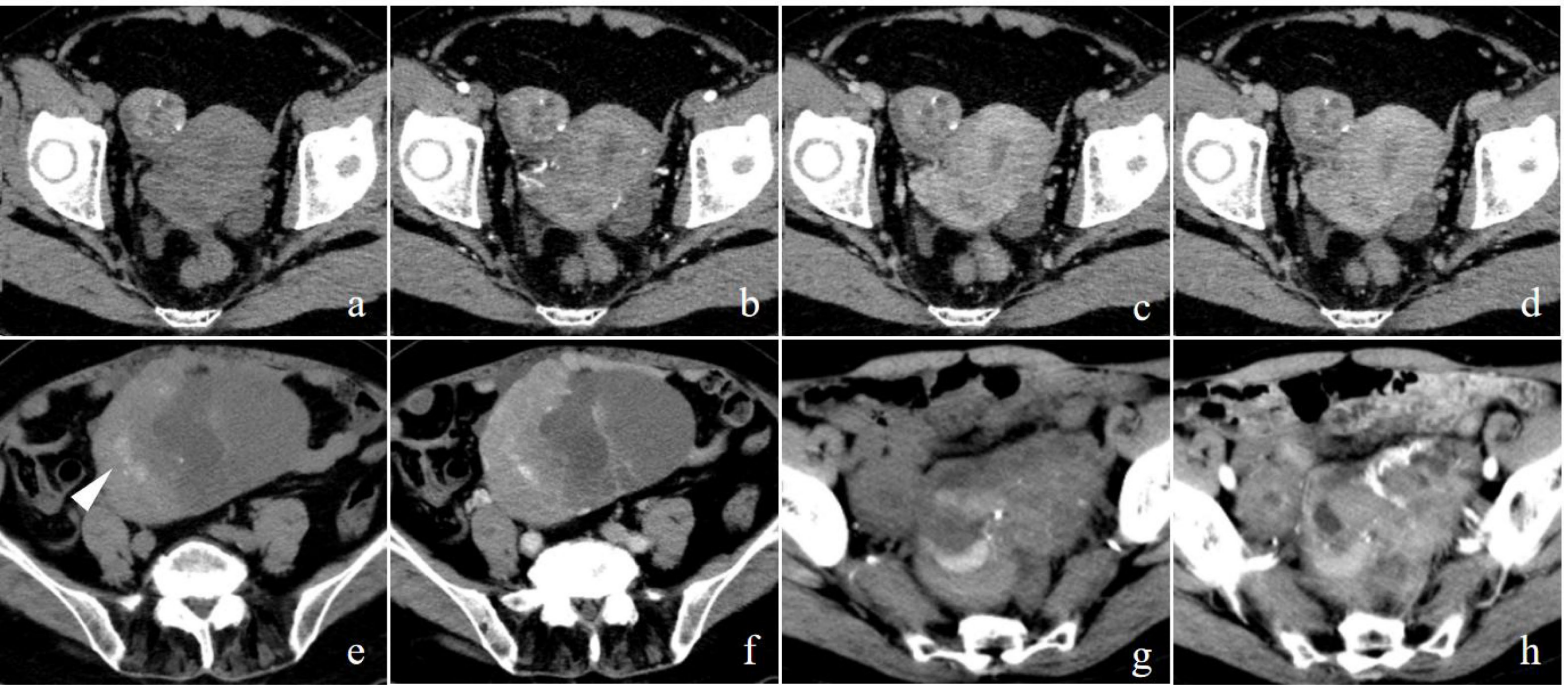
Three cases of BTs with CT and MRI scans. **(a–d)**. A 55-year-old woman diagnosed with a benign BT, characterized by a well-defined, mainly solid mass with irregular calcification and mild progressive enhancement on CECT scan. **(e, f)**. Borderline Brenner tumor in a 75-year-old woman. Amorphous clustered calcifications (arrow) and relatively prominent papillary projections are visible. **(g, h)**. A 71-year-old woman with a malignant BT, identified by an irregular mass containing hemorrhagic components, coarse calcifications, and pronounced enhancement.

**Figure 3:**
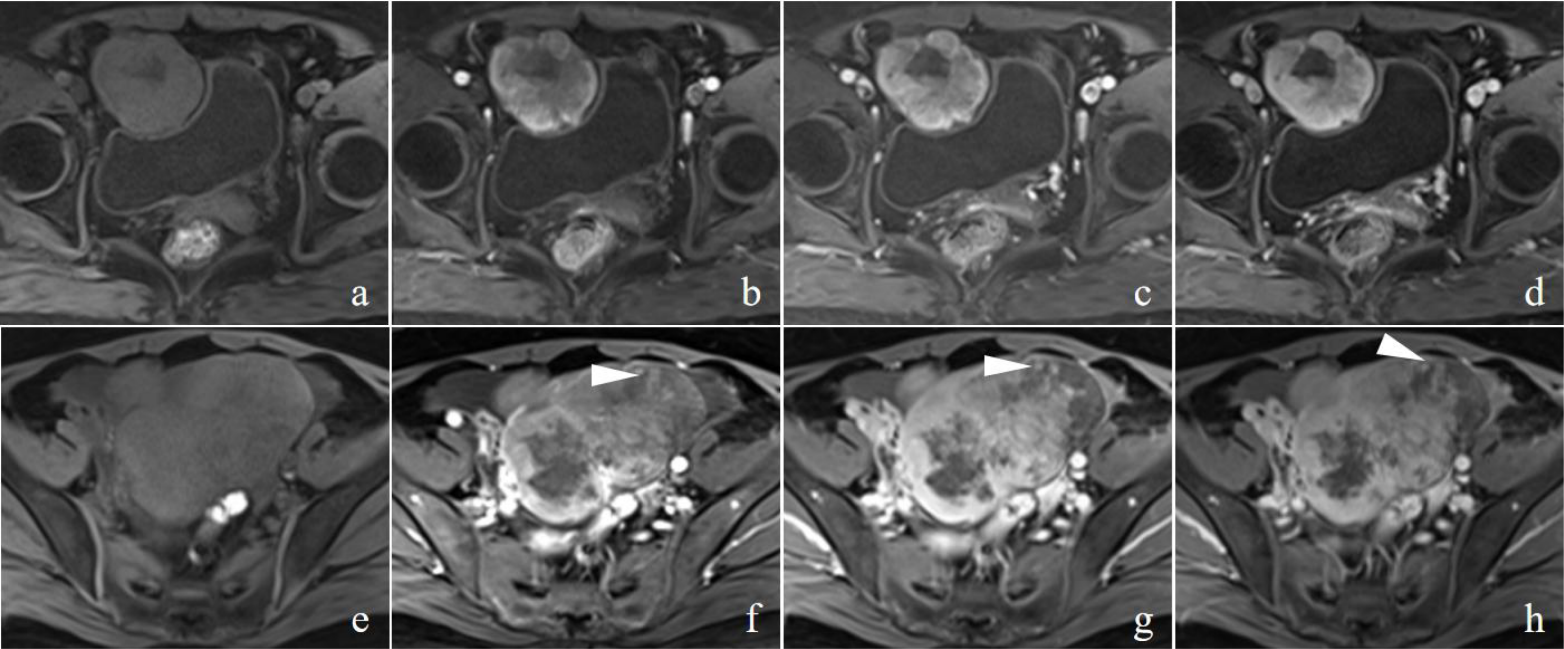
Enhancement patterns of benign, borderline, and malignant BTs. **(a–d)**. Benign BT exhibits progressive enhancement. In contrast, malignant BT **(e–h)** demonstrate persistent enhancement during the venous phase **(g)**, with contrast agent washout occurring in the delayed phase **(h)**. Papillary projections are indicated (arrowheads).

**Figure 4:**
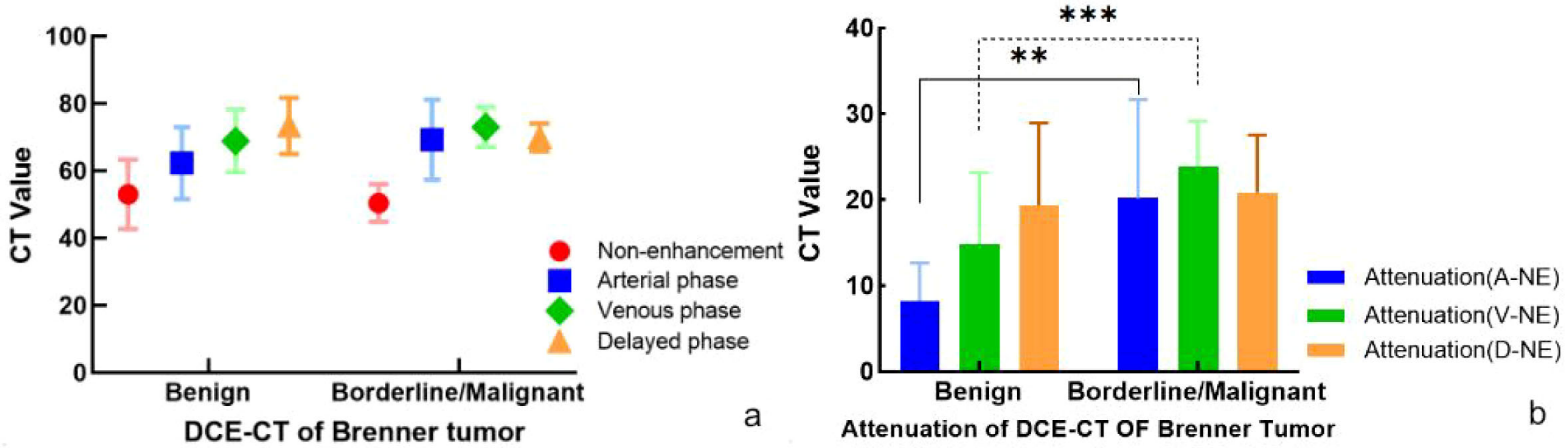
**(a)** Dynamic enhancement CT (DECT) demonstrates persistent enhancement in benign BTs, while borderline and malignant BTs show moderate to marked enhancement during the arterial and venous phases, followed by contrast agent washout in the delayed phase. **(b)** DECT indicates significant differences in attenuation values between the non-contrast and arterial phases or venous phases, as well as between the delayed phases, when comparing benign BTs to borderline and malignant BTs. ** indicates p < 0.01, and *** indicates p < 0.001.

The maximum diameter of benign BTs was observed to be 5.0(4.0–7.1)cm, whereas borderline/malignant BTs exhibited a larger maximum diameter of 8.0(7.5–12.1)cm, with this difference reaching statistical significance (p=0.001). Morphologically, benign BTs were predominantly cystic in nature, accounting for 80.5% (33/41) of cases, with a minority presenting as cystic-solid (19.5%, 8/41). Conversely, the majority of borderline/malignant BTs demonstrated a cystic-solid configuration (85.7%, 12/14), with only 14.3% (2/14) being predominantly cystic; this distribution was highly significant (p < 0.001). Ill-defined tumor margins were infrequent in benign BTs, observed in only 4.9% (2/41) of cases, whereas 35.7% (5/14) of borderline/malignant BTs exhibited ill-defined margins, a difference that was statistically significant (p=0.003). Furthermore, the presence of papillary projections served as a distinguishing characteristic, identified in 57.1% (8/14) of borderline/malignant BTs compared to only 2 cases in the benign group, with this difference also reaching high statistical significance (p<0.001). CT scans showed calcifications in 34 out of 36 BTs. Among benign tumors (n=25), 24 displayed calcifications, predominantly clustered irregular types (92.0%, 23/25), while scattered or punctate patterns were uncommon (4.0%, 1/25). In borderline or malignant tumors (n=11), 10 had calcifications, with clustered irregular types accounting for 63.6% (7/11) and scattered or punctate patterns for 27.3% (3/11). The distribution of calcification patterns differed significantly between groups (p = 0.03). Analysis of contrast-enhanced CT/MRI revealed that all 36 benign BTs with solid components exhibited mild to moderate enhancement, with no instances of marked enhancement (**Figures 2a–d, g, h**, **3a–d**). Among 13 borderline/malignant cases, 5 demonstrated marked enhancement (**Figures 2e, f, 3e–l**). The enhancement patterns differed significantly between the two groups (p < 0.001): benign BTs showed progressive enhancement, whereas borderline/malignant BTs reached peak enhancement during the venous phase followed by contrast washout in the delayed phase (**Figure 4a**). Quantitatively, benign BTs had CT values of 4.4 ± 1.9 HU between non-contrast vs arterial phase, 8.0 ± 3.5 HU between non-contrast vs venous phase, and 11.4 ± 3.1 HU between non-contrast vs delayed phase. Borderline/malignant BTs exhibited higher enhancement magnitudes, with net CT values of 20.6 ± 5.5 HU in the arterial phase, 25.2 ± 3.4 HU in the venous phase, and 22.5 ± 0.7 HU in the delayed phase (**Figure 4b**). The differences in CT attenuation values between the non-contrast and venous phases, as well as between the delayed phases, were found to be statistically significant when comparing benign and borderline/malignant groups (p < 0.05) see **Figures 2**–**4** and **Table 2**.

**Table 2:** CT/MRI findings of 55 lesions with benign and borderline/malignant BTs.

Image findings	Total n = 55 (%)	Benign n = 41 (%)	Borderline and malignant n = 14 (%)	*p* value
Maximum diameter (cm)	5.9 (4.1-8.0)	5.0 (4.0-7.1)	8.0 (7.5-12.1)	**0.001**
Location				0.95
Left	31 (56.4)	23 (56.1)	8 (57.1)	
Right	24 (43.6)	18 (43.9)	6 (42.9)	
Unilateral or bilateral				0.64
Unilateral	49 (89.1)	37 (90.2)	12 (85.7)	
Bilateral	6 (10.9)	4 (9.8)	2 (14.3)	
Shape				0.89
Irregular	25 (45.5)	18 (43.9)	7 (50.0)	
Round-like	30 (54.5)	23 (56.1)	7 (50.0)	
Appearance				<0.001
Cystic-solid	20 (36.4)	8 (19.5)	12 (85.7)	
Mainly solid	35 (63.6)	33 (80.5)	2 (14.3)	
Papillary projections	10 (18.2)	2 (4.9)	8 (57.1)	**<0.001**
Ill-defined boundaries	7 (12.7)	2 (4.9)	5 (35.7)	**0.003**
Pelvic effusion	27 (49.1)	20 (48.8)	7 (50.0)	0.94
CT values (Hu)				
NE	55.1 ± 6.5	55.6 ± 13.5	47.2 ± 3.5	0.26
A	64.9 ± 7.5	63.5 ± 13.8	71.5 ± 12.8	0.16
V	67.5 ± 4.5	69.4 ± 10.3	71.9 ± 6.5	0.35
D	70.0 ± 3.6	73.7 ± 8.7	69.9 ± 4.7	0.35
Attenuation (A-NE)	9.7 ± 9.7	4.4 ± 1.9	20.6 ± 5.5	**0.02**
Attenuation (V-NE)	12.4 ± 8.1	8.0 ± 3.5	25.2 ± 3.1	**<0.001**
Attenuation (D-NE)	14.9 ± 6.5	11.4 ± 3.1	22.5 ± 0.7	0.191
Calcifications (n=36)	36	25	11	0.03
Amorphous	30 (83.3)	23 (92.0)	7 (63.6)	
Punctate	4 (11.1)	1 (4.0)	3 (27.3)	
T1WI ratio (L/G)	0.92 ± 0.26	0.86 ± 0.17	1.02 ± 0.25	0.057
T2WI ratio (L/G)	0.98 ± 0.35	0.92 ± 0.32	1.12 ± 0.19	0.114
ADC ratio (L/G)	0.86 ± 0.32	0.76 ± 0.25	0.97 ± 0.37	0.096
ADC value	1330.4 ± 451.3	1378.0 ± 404.7	1211.5 ± 726.2	0.06
Enhanced pattern (n=49)				<0.001
Marked	5 (10.2)	0	5 (10.2)	
Moderate	13 (26.5)	8 (16.3)	5 (10.2)	
Mild	31 (63.3)	28 (57.2)	3 (6.1)	

NE, Non-enhanced CT scan; A, Arterial phase; V, Venous phase; D, Delayed phase. Bold values indicate statistical significance (p < 0.05).

The interpretation of imaging features demonstrated high inter-observer reliability and reproducibility. Specifically, quantitative parameters yielded excellent agreement, with ICCs of 0.97 (95% CI: 0.96–0.99) for maximum tumor diameter and 0.97 (95% CI: 0.95–0.99) for the venous-to-non-contrast (V–NE) CT attenuation difference. Consistent with these findings, categorical variables demonstrated substantial to excellent inter-observer agreement, as reflected by kappa values of 0.929 (95% CI: 0.815–0.957) for calcifications and 0.802 (95% CI: 0.705–0.922) for papillary projections. A comprehensive summary of both intra- and inter-observer analyses is available in **Table 3**.

**Table 3:** Intra- and interobserver analyses of quantitative data and categorical variables.

MRI/CT findings	Intra-observer				Inter-observer		
Quantitative variables	Observer 1	Observer 1	ICC	COV (%)	Observer 2	ICC	COV (%)
Maximum diameter	8.6 ± 4.5	8.3 ± 4.9	0.98 (0.96,0.99)	3.8	8.9 ± 5.6	0.97 (0.96,0.99)	3.1
CT values							
Attenuation (A-P)	6.0 (3.2-18.5)	6.2 (3.5-18.3)	0.99 (0.98,1.00)	4.1	5.6 (2.6-15.9)	0.96 (0.91,0.98)	4.7
Attenuation (V-P)	12.6 ± 9.1	12.9 ± 9.8	0.97 (0.96,0.99)	3.9	11.7 ± 8.3	0.97 (0.95,0.99)	5.1
Attenuation (D-P)	15.7 ± 7.7	14.8 ± 7.2	0.96 (0.92,0.99)	4.6	14.3 ± 5.1	0.99 (0.96,1.00)	4.2
MRI_ADC values	1311.6 ± 483.1	1353.8 ± 461.2	0.94 (0.85,0.97)	3.2	1339.9 ± 517.6	0.95 (0.89,0.97)	3.5
T1WI ratio (L/G)	0.9 ± 0.3	0.8 ± 0.2	0.95 (0.88,0.98)	4.0	0.9 ± 0.2	0.97 (0.94,0.99)	5.1
T2WI ratio (L/G)	0.8 ± 0.2	0.7 ± 0.3	0.92 (0.86,0.95)	3.6	0.7 ± 0.2	0.93 (0.84,0.95)	3.5
Categorical variables			Kappa			Kappa	
Location			0.85 (0.80-0.93)			0.88 (0.80-0.93)	
Unilateral or bilateral			0.92 (0.89-0.94)			0.92 (0.86-0.98)	
Shape			0.82 (0.76-0.89)			0.75 (0.71-0.84)	
Appearance			0.82 (0.73-0.89)			0.76 (0.71-0.90)	
Papillary projection			0.83 (0.75-0.91)			0.80 (0.71-0.92)	
Unclear boundary			0.83 (0.75-0.89)			0.74 (0.64-0.89)	
Pelvic effusion			0.78 (0.63-0.84)			0.79 (0.67-0.82)	
Calcification			0.94 (0.89-0.96)			0.93 (0.82-0.96)	
Enhancement type			0.86 (0.66-0.92)			0.79 (0.68-0.92)	

Data of ICC and Kappa are presented as value (95% confidence interval. Hu, Hounsfield Unit; A, Arterial phase; V, Venous phase; D, Delayed phase; ADC, Apparent diffusion coefficient; ICC, Intraclass Correlation Coefficient; COV, Coefficient of variation.

**Figure 5:**
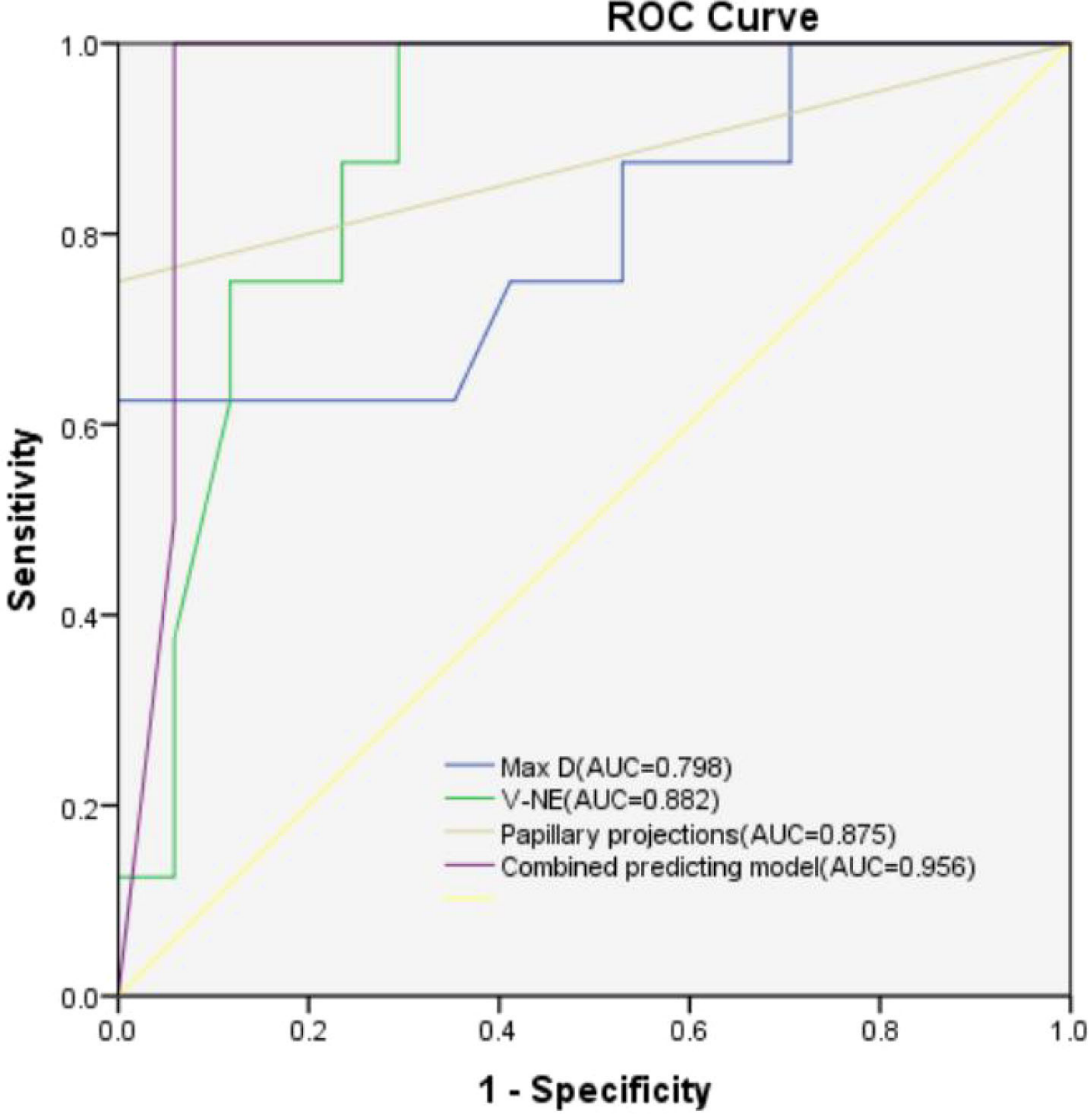
ROC curves of tumor maximum diameter, papillary features, venous-phase CT net enhancement, and their combined multivariate analysis in diagnosing borderline/malignant Brenner tumors (BTs).

Multivariate logistic regression analysis revealed that maximum tumor diameter, papillary projections, and venous-phase CT net enhancement are significant independent risk factors for distinguishing borderline from malignant BT (see **Table 4**). Individual multivariate analyses were created for each factor, as well as a combined model that included all three variables. The area under the curve (AUC) values for predicting BT characteristics were 0.789, 0.882, 0.875, and 0.956, respectively. The DeLong test showed that the combined model had the highest predictive accuracy for borderline versus malignant Brenner tumors (p < 0.001), with AUC values significantly higher than those of the other three models (all p < 0.001) (refer to **Table 4**; **Figure 5**).

**Table 4:** Multivariate logistic regression analysis.

Variables	Univariate OR (95% CI)	*p*	Multivariate OR (95% CI)	*B*	*p*
Maximum diameter	4.105 (4.796-18.243)	0.001	7.273 (3.325-42.625)	0.825	**0.03**
Appearance	1.156 (0.919-8.316)	0.04	16.037 (1.158-105.091)	1.405	0.07
Papillary projections	2.576 (0.885-7.492)	<0.001	0.022 (0.011-0.835)	0.883	**0.04**
Ill-defined boundaries	3.959 (1.265-18.729)	0.02	9.988 (0.431-231.589)	-1.296	0.34
Calcifications	2.236 (0.985-9.415)	0.03	16.037 (1.158-105.091)	-1.057	0.08
Enhanced pattern	5.592 (1.082-48.293)	<0.001	1.652 (0.065-5.742)	0.286	0.46
Attenuation (A-NE)	3.295 (2.288-19.242)	0.03	5.512 (3.069-115.048)	1.219	0.81
Attenuation (V-NE)	4.320 (2.481-53.122)	<0.001	6.954 (4.025-64.694)	1.704	**0.04**
CA125	0.236 (0.115-4.153)	0.03	0.175 (0.089-2.738)	0.542	0.27

OR, odds ratio; CI, confidence intervals. Bold values indicate statistical significance (p < 0.05).

### Pathological results

Among the 55 BTs examined, 41 (74.5%) were classified as benign, 10 (18.2%) as borderline, and 4 (7.3%) as malignant. Within the benign BT group, 13 cases (31.7%) were found to be associated with collision tumors, comprising 9 instances of mucinous cystadenoma (22%), 2 cases of serous cystadenoma (4.9%), and one case each of endometrioma (2.4%) and fibrothecoma (2.4%). In the combined borderline and malignant BT cohort (n=14), 5 cases (35.7%) exhibited collision tumors, including 2 cases of serous cystadenocarcinoma (14.3%), and one case each of mucinous cystadenoma (7.1%), serous cystadenoma (7.1%), and seromucinous cystadenoma (7.1%). Statistical analysis revealed no significant differences in either the type or frequency of collision tumors between the benign and borderline/malignant groups (p = 0.09).

### Initial preoperative imaging

Preoperative imaging correctly identified most benign Brenner tumors but often incorrectly diagnosed borderline or malignant tumors. Out of 41 benign tumors, there were 4 misdiagnoses (9.8%): one ovarian fibrothecoma, one malignant tumor, and two cystadenomas. For the 14 borderline or malignant tumors, 11 were misdiagnosed (78.6%), including one thecoma, three benign Brenner tumors, three endometrioid carcinomas, and four serous cystadenocarcinomas.

## Discussion

Ovarian BTs represent uncommon epithelial neoplasms of the ovary, histologically characterized by nests of urothelial cells embedded within a dense fibrous stroma (4, 9). Prior research has reported that approximately 95% of BTs are benign, while borderline and malignant variants constitute approximately 5% and 1%, respectively (10). Contrarily, a study encompassing 46 patients with BTs found that borderline tumors accounted for 2.2% and malignant tumors for 17.4% of cases (11). In the present study, the proportions of borderline and malignant tumors were observed to be as high as 18.2% and 7.3%, respectively. These findings suggest a potential increase in the incidence of borderline/malignant BTs. This variation highlights the critical clinical necessity for precise preoperative imaging standards to minimize misdiagnosis and improve surgical planning.

The preoperative misdiagnosis rate for borderline or malignant BTs remains as high as 78.6%, likely due to the rarity of the disease and limitations in conventional radiological interpretation. In our study, tumor maximum diameter, presence of papillary projections, and net enhancement on venous-phase CT were identified as independent risk factors for borderline or malignant BTs. Accordingly, the detection of a large mass with a distinctive fibrous framework, scattered calcifications, papillary projections, persistent venous-phase enhancement, and a delayed washout pattern may facilitate the diagnosis of these tumors. Although our proposed imaging features offer a preliminary step toward addressing this gap, further validation in larger cohorts is needed.

Tumor size is a conventional indicator for evaluating benign and malignant ovarian neoplasms. Previous research has established that the maximum diameter of malignant BTs is significantly greater than that of benign counterparts, suggesting that increased tumor volume may correlate with heightened cellular proliferative activity and malignant transformation (12, 13). Morphological features also provide key diagnostic clues. Benign BTs cells exhibit low proliferative activity and typically appear smooth, round, or glandular. In contrast, borderline and malignant BTs frequently present with distinct papillary projections. The underlying mechanism may be associated with uncontrolled cell proliferation and loss of normal apoptotic function. Abnormally proliferating cells continue to divide and grow into the cystic cavity or onto the tumor surface, forming papillary structures supported by fibrous connective tissue (14–17).

Prior studies have indicated that borderline and malignant BTs demonstrate greater enhancement than benign BTs (18, 19). However, precise CT distinctions remain poorly defined. Increased venous phase net enhancement was identified as an independent risk factor for borderline/malignant BTs. Benign BTs show gradual, sustained enhancement, whereas borderline/malignant BTs showed marked venous-phase enhancement followed by delayed washout (20, 21). Mechanistically, this difference may reflect stromal alterations: dense fibrotic stroma in benign BTs limits contrast diffusion, leading to gradual retention, whereas malignant transformation disrupts stromal integrity and promotes neoangiogenesis, explaining the venous-phase peak and delayed washout (22). This pattern differs from the rapid enhancement and early washout seen in ovarian cystadenocarcinomas, reflecting the more indolent behavior of borderline/malignant BTs and offering useful imaging distinctions between these ovarian lesions.

Clustered amorphous calcification is a distinctive imaging sign of BTs. In the current study, the prevalence of calcification detected by CT in BTs was 94.4%, which was slightly higher than the 70.4% reported in prior studies (23). Specifically, benign BTs predominantly exhibit extensive clustered amorphous calcifications, a characteristic attributed to the repeated deposition of calcium salts throughout the prolonged tumor growth period. Conversely, borderline and malignant BTs are primarily marked by punctate calcifications, likely resulting from the aggressive proliferation of malignant cells and heterogeneous calcium salt deposition (24, 25). By refining the calcification patterns of BTs, this study effectively enhanced the diagnostic accuracy for differentiating benign from malignant BTs.

BTs often occur alongside collision tumors, which makes clinical diagnosis more challenging (26). Borderline or malignant BTs typically have both cystic and solid parts, requiring careful differentiation between the tumor’s own structures and any coexisting collision tumors. These tumors are frequently mistaken for mucinous or serous cystadenocarcinomas. The presence of large, irregular calcifications within the tumor indicates a collision BT. Importantly, these calcifications are clearly different from the fine, sand-like calcifications commonly seen in serous cystadenocarcinomas, serving as a helpful feature to distinguish between these tumor types.

This study has several limitations. First, since it was a retrospective analysis and BTs are rare, this may have caused unavoidable statistical bias. Second, due to the small sample size (only 14 borderline/malignant lesions), the retrospective design, and lack of external validation, the reported AUC of 0.956 is probably overly optimistic. Therefore, the model’s generalizability cannot be confirmed and it needs to be validated in independent, prospective cohorts. Third, this research focused on imaging features to differentiate benign from borderline or malignant BT. However, in clinical practice, it is crucial to distinguish borderline/malignant BT from other types of ovarian cancer. Future research should therefore aim to differentiate BT from other ovarian tumors by integrating both clinical and imaging data.

In conclusion, larger tumor size, papillary projections, and venous-phase CT net enhancement independently distinguish borderline/malignant BTs from benign ones. Their distinctive venous-phase peak with delayed washout sets them apart from other ovarian cystadenocarcinomas, which may help improve preoperative risk assessment using pathology-confirmed cases.

## Data Availability

The original contributions presented in the study are included in the article/supplementary material. Further inquiries can be directed to the corresponding authors.

## References

[1] Chen M Liao S Cao Y Mao M Jia X Zhang S . Benign Brenner tumor of the ovary: two-dimensional and contrast-enhanced ultrasound features-a retrospective study from a single center. Front Oncol. (2024) 14:1337806. doi: 10.3389/fonc.2024.1337806 38525416 PMC10959004

[2] Costeira FS Félix A Cunha TM . Brenner tumors. Br J Radiol. (2022) 95:20210687. doi: 10.1259/bjr.20210687 34928171 PMC8822556

[3] Cree IA White VA Indave BI Lokuhetty D . Revising the WHO classification: female genital tract tumors. Histopathology. (2020) 76:151–6. doi: 10.1111/his.13977 31846528

[4] Jia Y Zhang S Bai F Zhu Z Li F Jia S . Imaging features and differential diagnosis of benign and borderline/malignant ovarian Brenner tumor. J Radiat Res Appl Sci. (2024) 17:100829. doi: 10.1016/j.jrras.2024.100829 38826717

[5] Nasioudis D Sisti G Holcomb K Kanninen T Witkin SS . Malignant brenner tumors of the ovary; a population-based analysis. Gynecol Oncol. (2016) 142:44–9. doi: 10.1016/j.ygyno.2016.04.538 27130406

[6] Turashvili G Hanley K . Malignant brenner tumor of the ovary: A critical reappraisal. Int J Gynecological Pathol. (2025) 44:182–92. doi: 10.1097/PGP.0000000000001060 39778112

[7] Sharma M Khangar B Mallya V Khurana N Gupta S . Coexisting brenner tumor and endometrial carcinoma. J Mid-life Health. (2017) 8:89. doi: 10.4103/jmh.JMH_3_17 28706410 PMC5496286

[8] Walter SR Dunsmuir WTM Westbrook JI . Inter-observer agreement and reliability assessment for observational studies of clinical work. J BioMed Inf. (2019) 100:103317. doi: 10.1016/j.jbi.2019.103317 31654801

[9] Kuhn E Ayhan A Shih I-M Seidman JD Kurman RJ . Ovarian Brenner tumor: A morphologic and immunohistochemical analysis suggesting an origin from fallopian tube epithelium. Eur J Cancer. (2013) 49:3839–49. doi: 10.1016/j.ejca.2013.08.011 24012099

[10] Roth LM Czernobilsky B . Ovarian brenner tumors. II. Malignant. Cancer. (1985) 56:592–601. doi: 10.1002/1097-0142(19850801)56:3<592::AID-CNCR2820560328>3.0.CO;2-A 4005816

[11] Yüksel D Kılıç C Çakır C Kimyon Cömert G Turan T Ünlübilgin E . Brenner tumors of the ovary: clinical features and outcomes in a single-center cohort. J Turkish German Gynecol Assoc. (2022) 23:22–7. doi: 10.4274/jtgga.galenos.2021.2021.0001 35000896 PMC8907439

[12] Silverberg SG . Brenner tumor of the ovary: A clinicopathologic study of 60 tumors in 54 women. Cancer. (1971) 28:588–96. doi: 10.1002/1097-0142(197109)28:3<588::AID-CNCR2820280310>3.0.CO;2-J 5096924

[13] Sagar K Lespinasse PF Haney A Conway NB . Brenner tumor of the ovary in a patient with postmenopausal bleeding: a case report. Cureus. (2024) 16(8):e67753. doi: 10.7759/cureus.67753 39318947 PMC11421885

[14] Saida T Yoshida M Shibuki S Ishiguro T Hoshiai S Sakai M . Comprehensive analysis of calcification frequency and patterns in ovarian tumors using non-contrast CT. Jpn J Radiol. (2025) 43:1153–65. doi: 10.1007/s11604-025-01750-4 40038215 PMC12204870

[15] Maturen KE Shampain KL Roseland ME Sakala MD Zhang M Stein EB . Malignant epithelial tumors of the ovary: pathogenesis and imaging. Radiol Clin North Am. (2023) 61:563–77. doi: 10.1016/j.rcl.2023.02.003 37169424

[16] Lang SM Mills AM Cantrell LA . Malignant brenner tumor of the ovary: review and case report. Gynecol Oncol Rep. (2017) 22:26–31. doi: 10.1016/j.gore.2017.07.001 28971141 PMC5608552

[17] Takahama J Ascher SM Hirohashi S Takewa M Ito T Iwasaki S . Borderline brenner tumor of the ovary: MRI findings. Abdom Imaging. (2004) 29:528–30. doi: 10.1007/s00261-003-0145-4 15024513

[18] Koufopoulos NI Pouliakis A Samaras MG Kotanidis C Boutas I Kontogeorgi A . Malignant brenner tumor of the ovary: a systematic review of the literature. Cancers. (2024) 16:1106. doi: 10.3390/cancers16061106 38539441 PMC10968811

[19] Matsutani H Nakai G Yamada T Yamamoto K Ohmichi M Osuga K . MRI and FDG PET/CT findings for borderline brenner tumor of the ovary: a case report and literature review. Case Rep Obstet Gynecol. (2020) 2020:8878649. doi: 10.1155/2020/8878649 32879741 PMC7448206

[20] Carmeliet P Jain RK . Angiogenesis in cancer and other diseases. Nature. (2000) 407:249–57. doi: 10.1038/35025220 11001068

[21] Jackson A O’Connor JPB Parker GJM Jayson GC . Imaging tumor vascular heterogeneity and angiogenesis using dynamic contrast-enhanced magnetic resonance imaging. Clin Cancer Res Off J Am Assoc Cancer Res. (2007) 13:3449–59. doi: 10.1158/1078-0432.CCR-07-0238 17575207

[22] Thomassin-Naggara I Cuenod CA Darai E Marsault C Bazot M . Dynamic contrast-enhanced MR imaging of ovarian neoplasms: current status and future perspectives. Magn Reson Imaging Clin N Am. (2008) 16:661–72. doi: 10.1016/j.mric.2008.07.012 18926429

[23] Montoriol P-F Hordonneau C Boudinaud C Molnar I Abrial C Kossai M . Benign Brenner tumor of the ovary: CT and MRI features. Clin Radiol. (2021) 76:593–8. doi: 10.1016/j.crad.2021.03.018 33933275

[24] Famenini S Cassarino DS . Dermatofibroma‐associated dystrophic calcification. J Cutan Pathol. (2014) 41:68–70. doi: 10.1111/cup.12255 24224946

[25] Parcesepe P Coppola L Remo A D’Andrea MR Coppola G Simbolo M . Molecular and clinical insights in Malignant brenner tumor of the testis with liver metastases:A case report. Front Oncol. (2021) 11:663489. doi: 10.3389/fonc.2021.663489 33912469 PMC8072450

[26] Pavlovic A Glavina Durdov M Lozic D Skare Librenjak L Alfirevic D . Primary ovarian lymphoma and benign brenner tumor. Taiwan J Obstet Gynecol. (2016) 55:138–9. doi: 10.1016/j.tjog.2015.04.005 26927268

